# Gut microbiota composition and systemic immune-inflammatory marker correlations in infertile women with endometriosis: a pilot case–control study

**DOI:** 10.3389/fcimb.2026.1720894

**Published:** 2026-01-23

**Authors:** Xiaoli Dong, Xiaozhen Chen, Yingpei Xu, Defei Zeng, Ping Li

**Affiliations:** 1 Reproductive Medicine Center, Longyan First Affiliated Hospital of Fujian Medical University, Longyan, Fujian, China; 2 Gastrointestinal Endoscopy Center, Longyan First Affiliated Hospital of Fujian Medical University, Longyan, Fujian, China

**Keywords:** endometriosis, gut microbiome, gut–immune axis, interleukin-6, macrophage migration inhibitory factor, short-chain fatty acids, tumor necrosis facto

## Abstract

**Background:**

The specific gut microbial signatures and their correlation with immune-inflammatory markers in infertile women with endometriosis remain underexplored.To investigate the differences in gut microbiota and their associations with biochemical immune markers in infertile women with endometriosis compared to controls.

**Methods:**

This case-control study enrolled 32 infertile women with endometriosis and 13 control women with male-factor infertility. Fecal samples were collected for 16S rRNA sequencing to profile the gut microbiota, and serum samples were obtained to measure inflammation-related biomarkers. Bioinformatics analyses were applied to compare gut microbial community structures and to examine correlations between differentially abundant bacteria and immune markers.

**Results:**

The endometriosis group exhibited significant enrichment of Lachnospira, Bacilli, Lactobacillales, Parasutterella, Enterococcus, and Veillonella. Comparative analysis revealed significantly altered abundances of multiple taxa, including Lachnospira, Parasutterella, Alistipes, Enterococcus, Veillonella, Streptococcus, Desulfovibrionaceae, Ruminococcaceae, Bilophila, and Peptoniphilus (all P < 0.05). Several inter-species correlations were identified among these bacteria. Importantly, specific microbiota were correlated with immune markers: Streptococcus and Veillonella were positively correlated with macrophage migration inhibitory factor (MIF); Bilophila and Enterococcus were positively correlated with TNF-α and IL-6; Veillonella was positively correlated with TNF-α; Desulfovibrionaceae was negatively correlated with TNF-α and IL-6; and Parasutterella was negatively correlated with CA125.

**Conclusion:**

In this exploratory investigation, specific gut microbial signatures were observed in infertile patients with endometriosis, showing correlations with select systemic immune-inflammatory biomarkers. These initial observations point to a possible association between gut microbiota imbalance and the inflammatory aspects of endometriosis-associated infertility. Consequently, microbial modulation merits further investigation as a potential strategy to alleviate inflammation and potentially enhance reproductive outcomes.

## Introduction

Endometriosis (EMs) is a common gynecological disorder affecting women of reproductive age and represents a major contributor to female infertility manifestations often include dysmenorrhea, chronic pelvic pain, and impaired fertility (Chou et al., 2021; Zheng et al., 2023). Epidemiological data indicate that EMs affects approximately 10–15% of reproductive-aged women, translating to an estimated 176 million cases globally. Notably, endometriosis is present in about 40% of infertile patients (Khan, 2020; Corachán et al., 2021; Lindheim et al., 2024). The condition is frequently characterized by subtle and nonspecific symptoms, leading to diagnostic delays that may exacerbate disease progression, complicate treatment, and adversely affect long-term outcomes.

The etiology of endometriosis remains incompletely understood (Mikhaleva et al., 2021; Tennfjord et al., 2021). Its invasive and recurrent behavior, along with its anatomical proximity to the intestinal tract, has drawn comparisons to malignant tumors. In recent years, growing evidence has highlighted a significant association between EMs and gut microbiota homeostasis (Tang et al., 2024). Advances in genomic sequencing, particularly 16S rRNA sequencing, have enabled detailed phylogenetic analysis of microbial communities, greatly facilitating comprehensive microbiome research (Wensel et al., 2022; Yang et al., 2024). The gut–reproductive axis has been increasingly recognized as a contributor to female reproductive health. Gut microbiota can modulate estrogen metabolism via the estrobolome, influence systemic and local immune responses, and shape low-grade chronic inflammation, all of which are relevant to endometriosis and infertility. However, most human studies have focused on pelvic or endometrial microbiota or on non-infertile women, and few have simultaneously examined gut microbiota and systemic immune-inflammatory markers in infertile women with endometriosis.

Emerging studies further suggest that endometriosis may represent a systemic chronic inflammatory condition (Gajbhiye, 2023). Key inflammatory mediators such as macrophage migration inhibitory factor (MIF), tumor necrosis factor-alpha (TNF-α), and interleukin-6 (IL-6) have been implicated in its pathogenesis (Mohammed Rasheed and Hamid, 2020). However, the roles of these biomarkers in infertile women with endometriosis remain underexplored.

In this study, we enrolled infertile women diagnosed with endometriosis and a control group of women with infertility due to male factors. Fecal and serum samples were collected from all participants. Using 16S rRNA next-generation sequencing, we analyzed the gut microbiota composition and identified differential microbial profiles between the two groups. Our objective was to pinpoint characteristic bacterial communities enriched in endometriosis-associated infertility, which may offer potential diagnostic or therapeutic targets. Additionally, serum levels of MIF, TNF-α, and IL-6 were quantified via enzyme-linked immunosorbent assay. We further investigated the correlation between the relative abundance of specific gut bacteria and circulating inflammatory marker levels in infertile women with endometriosis. The findings are presented in the subsequent sections.

## Materials and methods

### Study population

This exploratory, pilot case-control study was designed to investigate initial correlations, acknowledging limitations inherent to its preliminary nature. Sample size was determined by the number of eligible infertile women with surgically or sonographically confirmed endometriosis who presented during the defined study period; a formal *a priori* power calculation was not performed due to the exploratory design. The study cohort comprised 32 women with endometriosis-related infertility and 13 control women with infertility attributed solely to a male factor. Women with male-factor infertility were selected as a clinically relevant reference group, as they undergo equivalent comprehensive gynecological and sonographic evaluations yet present without clinical or imaging evidence of uterine, ovarian, or pelvic pathology. The recruitment of truly healthy, parous women as controls was not ethically or practically feasible within the context of this infertility clinic setting. All participants were sampled during the follicular phase of the menstrual cycle (confirmed by self-report and serum hormone levels). None had used antibiotics or probiotics for at least three months prior to sample collection. Basic dietary patterns were assessed via a food frequency questionnaire, and no significant differences in major dietary components were observed between the two groups.All of whom were treated at the Reproductive Medicine Center of Longyan First Hospital between July 2022 and December 2023. Women with endometriosis-related infertility must be diagnosed with ovarian endometriosis through ultrasound or surgery.Clinical data collected included general examinations, age, duration of infertility, marital and reproductive history, medical history, menstrual history, dysmenorrhea symptoms, pathological diagnoses, lab test results, and other diagnostic information. Data from the health check-up group was also gathered, including age, lab results, and diagnostic information. This study was approved by the Ethics Committee of Longyan First Hospital (No. LYREC2022-010-01, approval date 21 June 2022). All participants provided informed consent and signed consent forms.

Inclusion criteria: Age < 35 years; body mass index (BMI) between 18 and 24 kg/m²; not pregnant; no use of systemic antibiotics, probiotics, or immunosuppressive drugs within the preceding three months.; no history of smoking, excessive alcohol consumption, or drug abuse.

Exclusion criteria: Polycystic ovary syndrome; cervical HPV infection or cervical abnormalities; acute reproductive or urinary tract infections; or serious internal or external health conditions.

### Sample collection

All fecal and blood samples were collected between days 2 and 4 of the menstrual cycle in both groups to minimize hormonal variability. Participants were instructed to collect a fresh stool sample in a sterile container in the morning after an overnight fast. Samples were transported to the laboratory within 4 hours in an insulated box with ice packs and immediately stored at −80 °C until DNA extraction. These samples will be used for 16S rRNA sequencing analysis of the gut microbiota and detection of serum MMIF, TNF-α, and IL-6. DNA concentration and purity were assessed using a NanoDrop spectrophotometer, and samples with OD260/280 between 1.8 and 2.0 were included. DNA integrity was verified by agarose gel electrophoresis.

### Bacterial 16S rRNA gene amplicon sequencing

DNA was extracted from swabs using the Magnetic Soil and Stool DNA Kit for 16S rRNA gene sequencing. The V1-V2 region of the 16S rRNA gene was amplified with the 27F (5’-AGAGTTTGATCCTGGCTCAG-3’) and 338R (5’-TGCTGCCTCCCGTAGGAGT-3’) primers. The V1–V2 region was chosen because it has been extensively validated in our laboratory and offers good resolution for Firmicutes/Bacteroidetes taxa on the Illumina NovaSeq platform. However, compared with the more commonly used V3–V4 region, V1–V2 may provide different taxonomic coverage and complicate direct comparisons across studies, which should be taken into account when interpreting our findings. The PCR products were pooled in equal amounts and purified using the GeneJET Gel Extraction Kit. Negative controls were included during DNA extraction and PCR amplification to monitor potential contamination. ASVs predominantly present in negative controls or known reagent contaminants were identified and removed during downstream analysis. Sequencing libraries were prepared using the TruSeq DNA PCR-Free Sample Preparation Kit (Illumina) according to the manufacturer’s instructions. The libraries were sequenced on the Illumina NovaSeq 6000 platform, generating 250-bp paired-end reads (Novogene). Sequencing data were processed using the Quantitative Insights Into Microbial Ecology 2 (QIIME2 2022.2) pipeline. Single-end sequences were filtered and denoised using the DADA2 plugin, resulting in 6, 510 amplicon sequence variants (ASVs). Taxonomy was assigned to the ASVs using the q2-feature-classifier classify-sklearn naive Bayes taxonomy classifier, referencing the RDP v18 ribosomal RNA gene database. ASVs were inferred using the DADA2 plugin in QIIME2 (version 2022.2) with the following parameters: truncLen = 240–260 bp, maxEE = 2, and removal of chimeric sequences using the consensus method. After quality filtering, denoising, and chimera removal, a total of 1, 004, 637 high-quality reads were obtained (median 49, 942 reads per sample, interquartile range 43, 391–55, 553), corresponding to 3, 372 ASVs. Rarefaction curves were generated to evaluate sequencing depth and indicated that a rarefaction depth of 30, 000 reads per sample provided adequate coverage for alpha-diversity analyses.

### Statistical analysis

Statistical analysis was performed using SPSS 23.0. Continuous variables that were normally distributed with equal variances were analyzed using independent t-tests. For non-normally distributed data, the Kruskal-Wallis rank-sum test was applied. Comparisons of categorical data rates were made using the χ² test or Fisher’s exact test. Alpha diversity indices were calculated at the chosen rarefaction depth using QIIME2. Beta diversity was assessed using Bray–Curtis dissimilarity and weighted and unweighted UniFrac distances, and differences in overall community structure between groups were tested with PERMANOVA (999 permutations). Ordination was visualized using principal coordinates analysis (PCoA). Differences in microbiota between groups were analyzed using principal component analysis. LEfSe analysis was used as an exploratory tool to identify taxa differentially enriched between groups, with an LDA score threshold of 2.0. The Kruskal–Wallis P values underlying the LEfSe results are reported in **Supplementary Table 1** together with FDR-adjusted q values. P values from multiple univariate comparisons of bacterial taxa between groups were adjusted for multiple testing using the Benjamini–Hochberg false discovery rate (FDR) procedure. Taxa with FDR-adjusted q < 0.1 were considered statistically significant.

## Results

### Characterization of the study cohort

This study included a total of 45 female infertility participants, 32 of whom had endometriosis (EMs group), and 13 had male factor infertility (Control group). The clinical characteristics and biochemical markers of the EMs group and the control group are summarized in **Table 1**. To compare and analyze the differences between the two groups, several variables were assessed. There were no significant differences between the groups in terms of age, duration of infertility, and anti-Mullerian hormone(AMH) (P>0.05). However, BMI was significantly lower in the EMs group compared to the control group (P<0.05). Notably, serum levels of cancer antigen 125(CA125), MMIF, and TNF-a were significantly higher in the EMs group compared to the control group (P<0.05). On the other hand, IL-6 levels did not show a significant difference between the two groups (P<0.05).

**Table 1:** Comparison of baseline characteristics between endometriosis group and control group.

Variable	EMs group (n=32)	Normal group (n=13)	P-value
Age (years)	31.00 ± 4.08	31.85 ± 3.11	P>0.05
Body mass index (kg/m2)	20.94 ± 2.17	22.35 ± 1.94	P<0.05
Duration of infertility (year)	2.00(1.00, 4.00)	3.00(2.00, 7.50)	P>0.05
AMH (ng/mL)	2.97(2.13, 4.65)	3.52(2.23, 4.04)	P>0.05
CA125 (U/ml)	40.50(23.25, 63.88)	21.09(13.70, 30.35)	P<0.05
MMIF (ug/L)	96.27(81.61, 185.82)	59.49(48.54, 74.69)	P<0.05
TNF-a (pg/mL)	4.84(3.67, 10.06)	3.69(3.10, 4.78)	P<0.05
IL-6 (pg/mL)	2.00(1.47, 3.57)	1.99(1.51, 2.32)	P>0.05

Data presented as median (interquartile range) or n (%).

a Mann Whitney test.

b Chi-squared test.

AMH, anti-Mullerian hormone.

### Analysis of gut microbiota diversity in different groups

**Figure 1:**
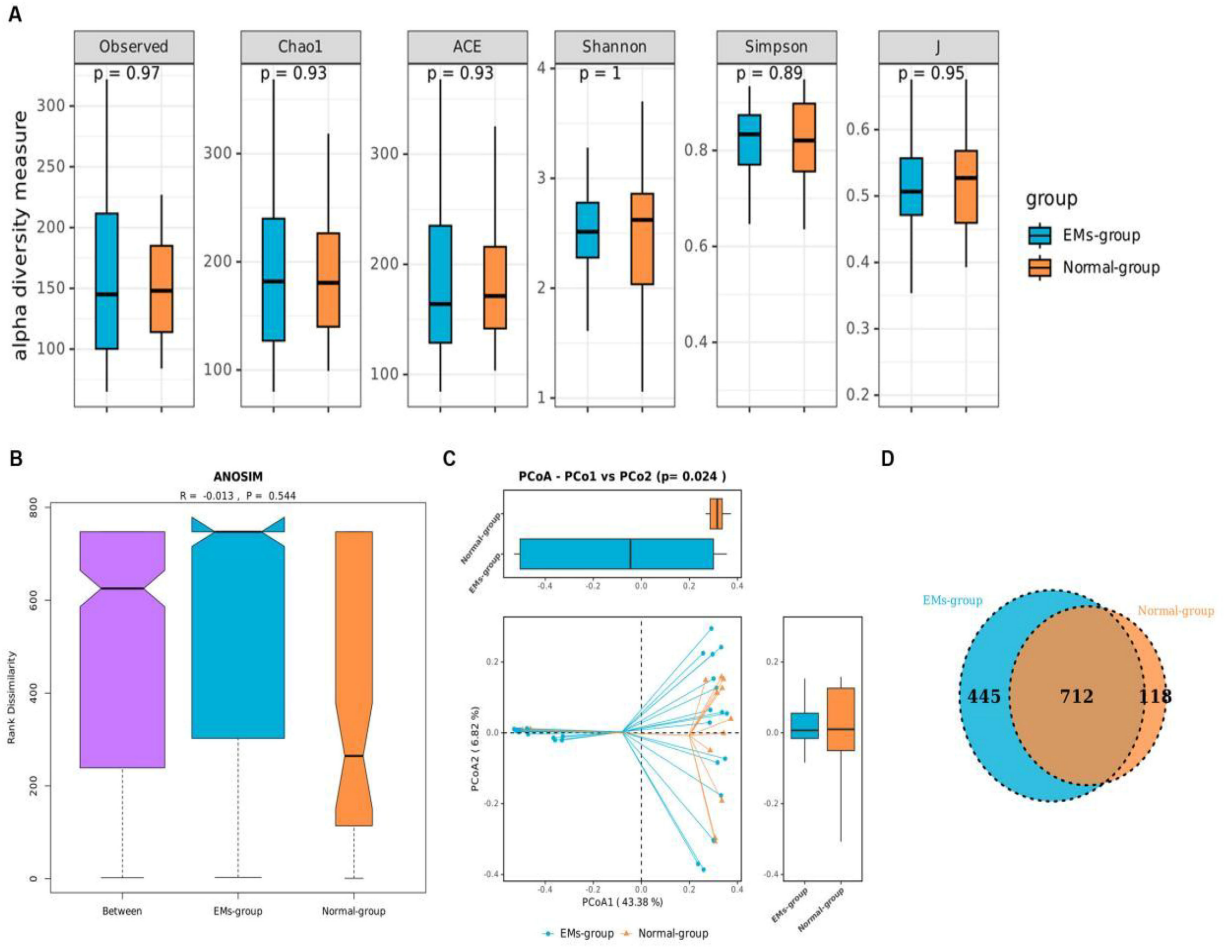
Analysis of gut microbiota diversity in different groups. **(A)** The α-diversity indices were used to analyze the complexity of the microbial community composition within the samples, reflecting the richness (ACE, Chao1, Observed species), diversity (Shannon and Simpson), and evenness (J) of the microbial communities. The x-axis represents the sample groups, while the y-axis shows the alpha diversity indices. **(B)** Analysis of gut microbiota community similarity between different groups. The x-axis shows all samples (Between) and individual groups, while the y-axis represents the rank of distance. If the Between group rank is higher than that of other groups, it suggests that the between-group differences are greater than the within-group differences. R ranges from -1 to 1; when R > 0, it indicates significant between-group differences, and when R < 0, it suggests within-group differences are greater than between-group differences. If the troughs of two groups do not overlap, it indicates a significant difference in their medians. The statistical significance is represented by P, with P < 0.05 indicating significance. **(C)** A comparative analysis of the microbial community structure across different groups. Each point in the figure represents a sample, with different colors indicating different experimental groups. The closer the points are to each other, the more similar the samples are. PCoA (Principal Coordinate Analysis) is performed using a distance matrix calculated from the species composition of the samples. The x-axis and y-axis represent the contribution of the first (PC1) and second (PC2) principal components, respectively. **(D)** The similarity in OTU composition between different groups. Different colors represent samples from different groups, with the numbers in the middle indicating the number of OTUs in each color block. The overlapping areas of the color blocks represent shared OTUs.

We evaluated sequencing coverage using rarefaction curves (**Supplementary Figure 1**); all samples plateaued at 30–000 reads, indicating adequate depth for downstream diversity analyses. Then, we compared the α-diversity of the gut microbiota between the two groups using the Observed, Chao1, ACE, Shannon, Simpson, and J indices. The results showed no statistically significant differences (P>0.05), indicating that the gut microbiome diversity was similar between the two groups (**Figure 1A**). A similarity analysis (ANOSIM) was conducted to compare within-group and between-group similarities based on distance metrics, testing the null hypothesis that the average rank similarity between samples within the same group is equal to the average rank similarity between samples from different groups. No significant differences were found in the bacterial distribution between the two groups (P>0.05) (**Figure 1B**). Principal Coordinates Analysis (PCoA) was performed using a distance matrix calculated from the species composition of the samples. The x-axis and y-axis represent the contributions of the first principal component (PCoA1) at 43.38% and the second principal component (PCoA2) at 6.82%, respectively (p=0.024) (**Figure 1C**). The Venn diagram illustrates the common and unique OTUs between the gut microbiota of the two groups. The size of the circles represents the number of OTUs. The EMs group had 712 common OTUs and 445 unique OTUs, while the control group had 118 unique OTUs (**Figure 1D**).

### Distribution of gut microbiota communities in different groups

**Figure 2:**
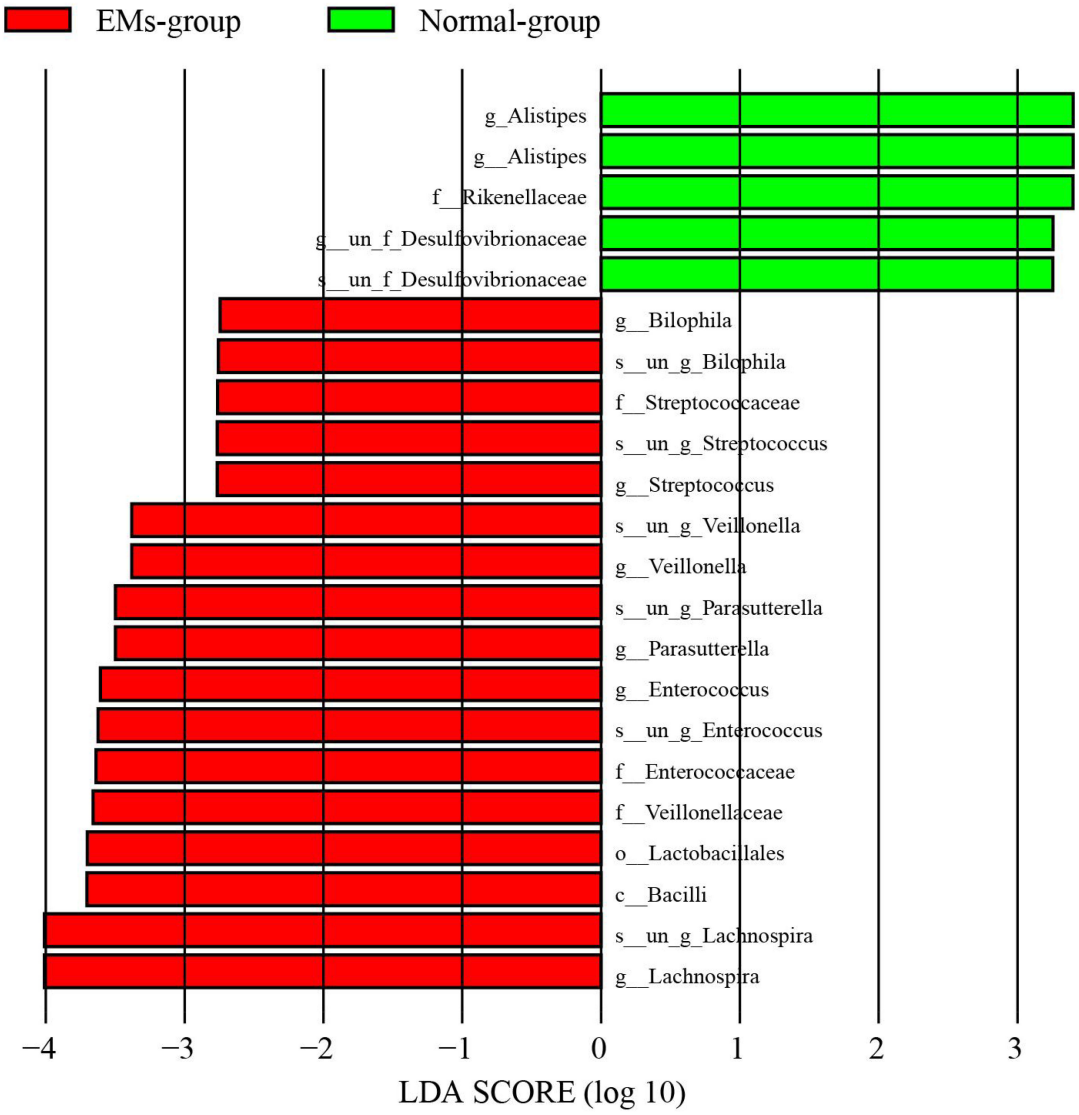
Differential species analysis between groups. The LDA score distribution plot shows species with an LDA score greater than the set threshold, indicating statistically significant biomarkers. It also displays species with significant abundance differences between groups, with the length of the bars representing the magnitude of the impact of these significantly different species.

LefSe (Linear Discriminant Analysis Effect Size, LDA Effect Size) is a tool commonly used to analyze group differences and identify specific microbial species. It’s useful for developing biomarkers and supporting further research. For two group, we set the LDA threshold at 2 to identify characteristic bacterial communities for each group. We found twelve microbial taxa with significant differences in the EMs group and five in the control group (**Figure 2**). Specifically, Lachnospira, Bacilli, Lactobacillales, Parasutterella, Enterococcus and Veillonella were more abundant in the EMs group, while Alistipes, Rikenellaceae and Desulfovibrionaceae were more abundant in the control group. All species selected by LEfSe were further subjected to FDR correction (**Supplementary Table 1**).

### Comparison of gut microbiota abundance between the two groups

**Figure 3:**
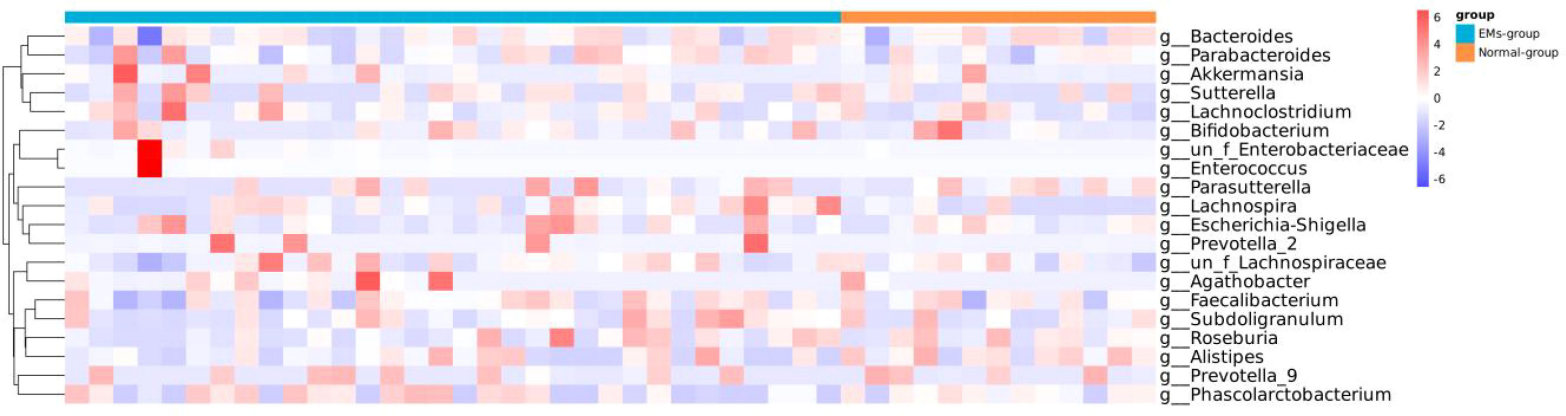
A comparison of the abundance of the top 20 dominant species at the genus level between samples. Different colors represent the relative abundance of different species. The similarity relationships between species are shown on the left side of the figure.

**Figure 4:**
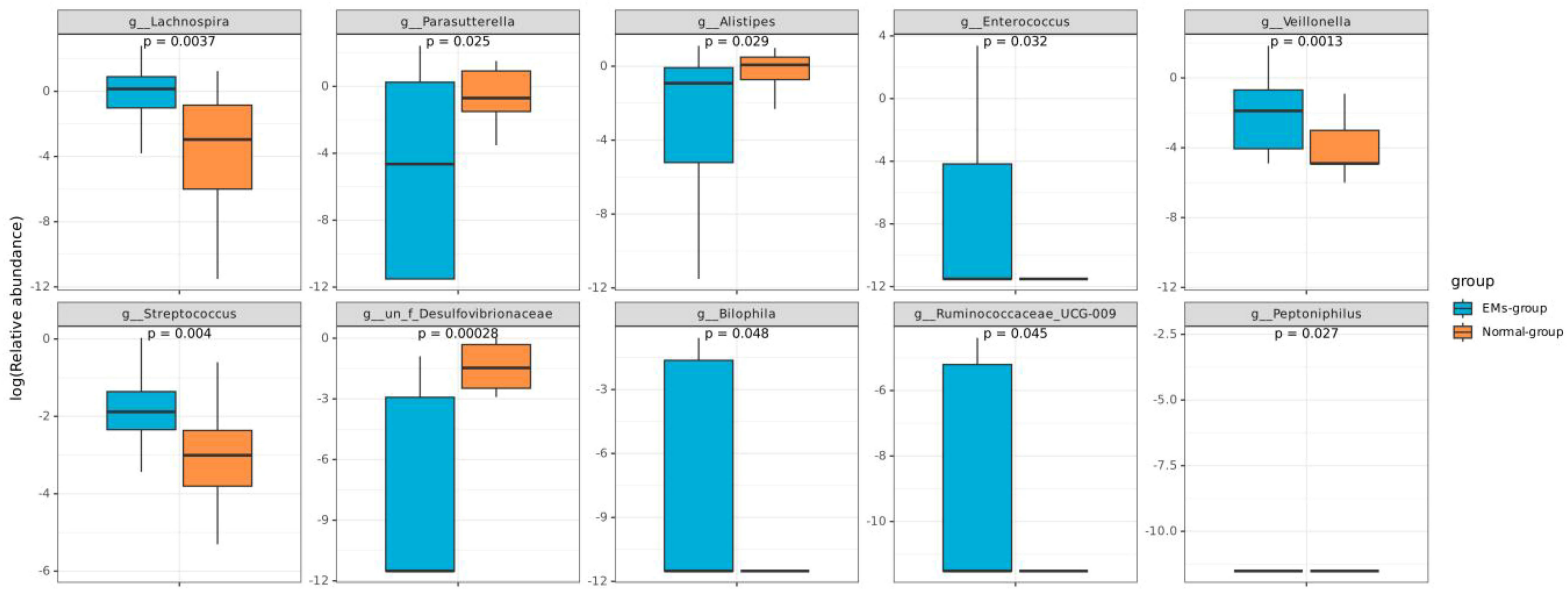
The distribution of relative abundance of gut microbiota at the genus level. It highlights significant differences in abundance between groups and lists the top 10 species with the highest average abundance.

**Figure 5:**
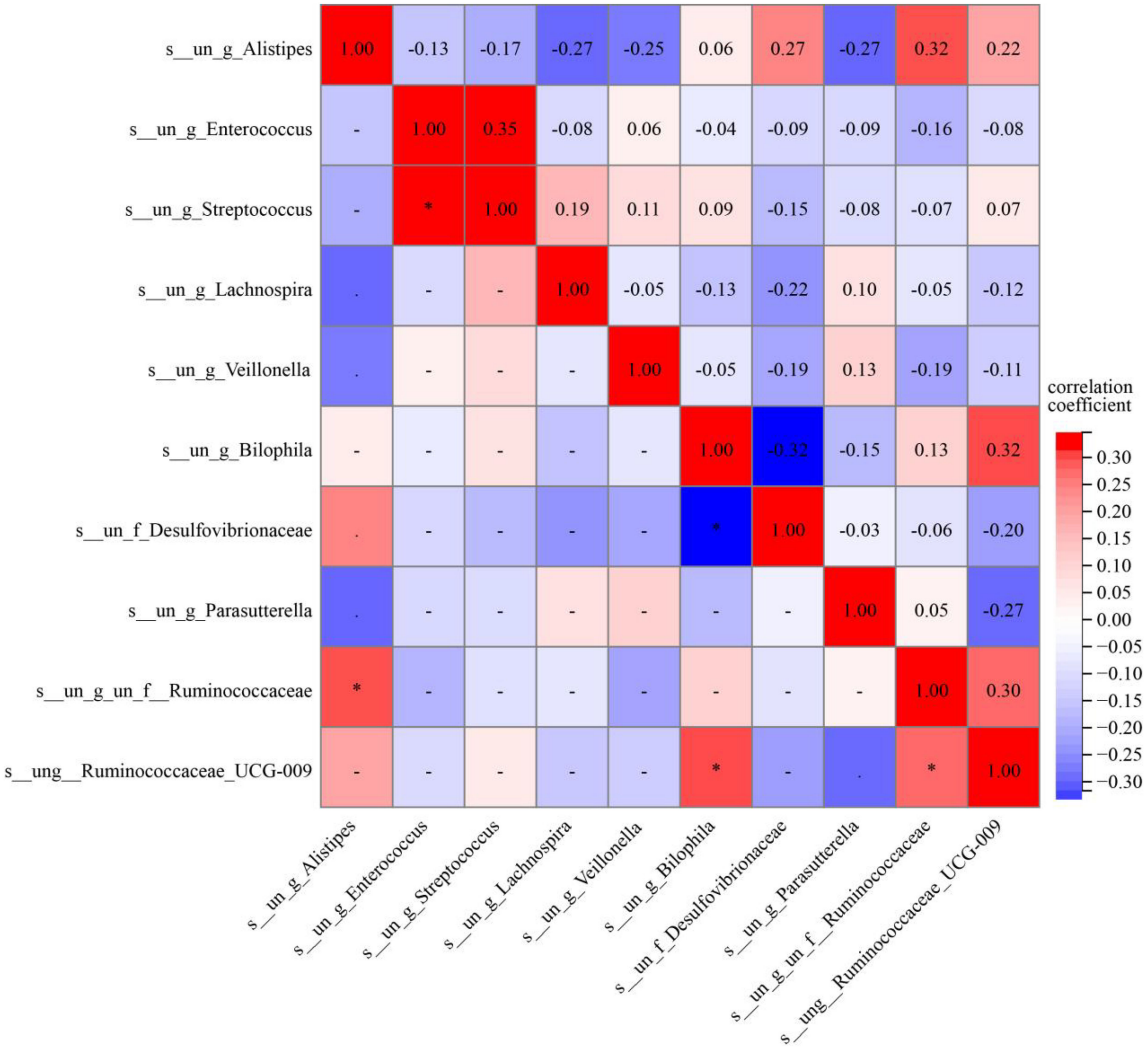
This shows the significant abundance differences between groups and the correlations of the top 10 species with the highest average abundance. Red represents a positive correlation, while blue represents a negative correlation (p<0.001: ***; p<0.01: **; p<0.05: *).

We analyzed the clustering of the top 20 dominant species at the genus levels to compare bacterial abundance between the two groups (**Figure 3**). The Wilcoxon signed-rank test and Kruskal-Wallis rank-sum test were used to identify species with significant differences in abundance between the groups. Our analysis revealed significant differences in the abundance of Lachnospira, Parasutterella, Alistipes, Enterococcus, Veillonella, Streptococcus, Desulfovibrionaceae, Ruminococcaceae, Bilophila and Peptoniphilus between the two groups (P<0.05) (**Figure 4**). The P-values after FDR correction are provided in **Supplementary Table 2**. To further explore the relationship among species with significant abundance differences, we conducted a Pearson correlation analysis. The results indicated that Desulfovibrionaceae was negatively correlated with Bilophila (P<0.05), and in addition, Ruminococcaceae_UCG-009 was positively correlated with Bilophila, Alistipes, and Ruminococcacceae (P<0.05) (**Figure 5**).

### Spearman correlation analysis between gut microbiota and biochemical immune markers

**Figure 6:**
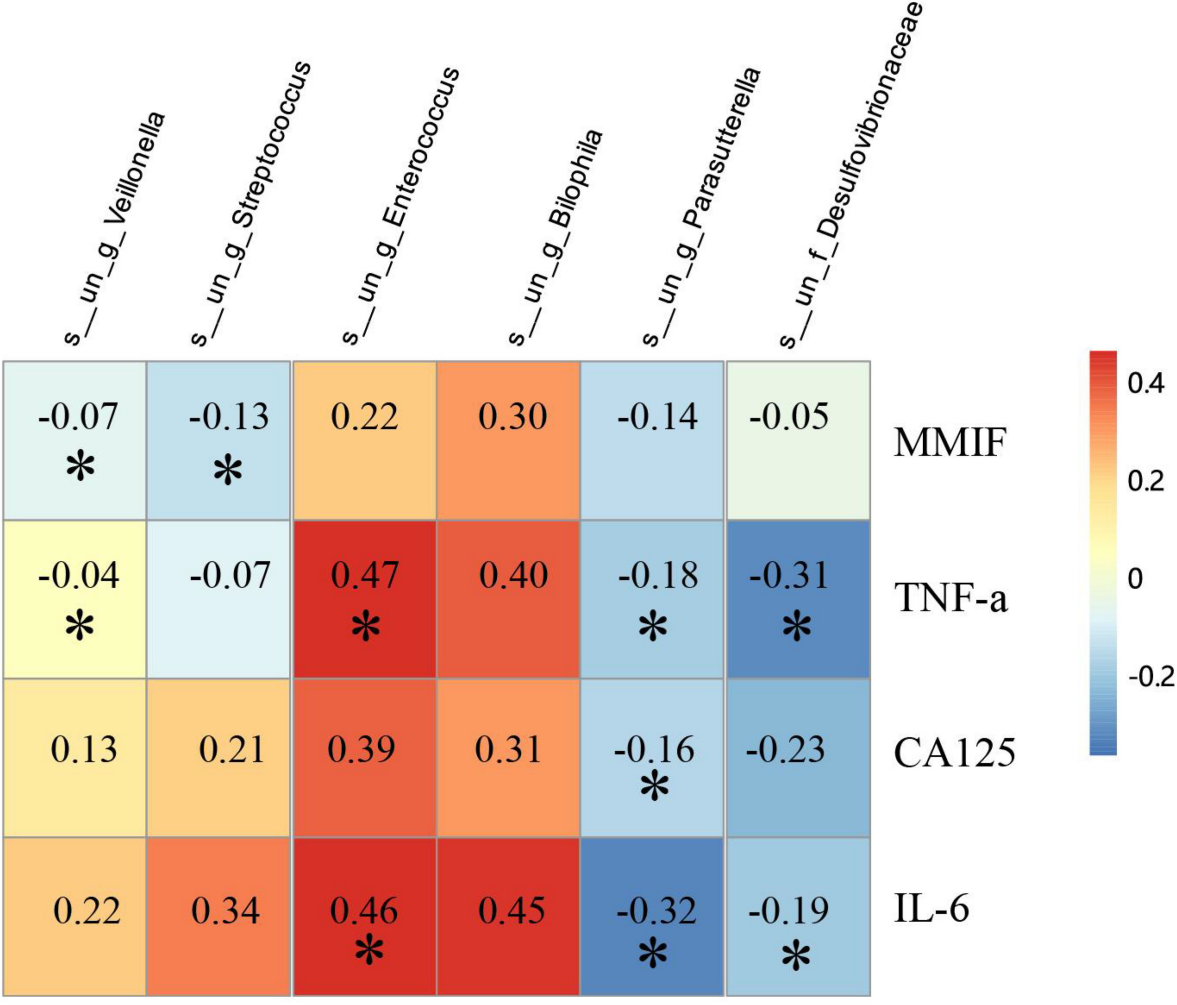
Correlation analysis of MMIF, TNF-α, IL-6 and CA125 with gut microbiota. Red represents a positive correlation, while blue represents a negative correlation (p<0.001: ***; p<0.01: **; p<0.05: *).

The study revealed the following correlations: Streptococcus and Veillonella were positively correlated with MMIF (P<0.05). Both Bilophila and Enterococcus showed a positive correlation with TNF-α and IL-6 (P<0.05), while Veillonella was positively correlated with TNF-α (P<0.05). Desulfovibrionaceae had a negative correlation with both TNF-α and IL-6 (P<0.05). Additionally, Parasutterella was negatively correlated with CA125 (P<0.05) (**Figure 6**).

Multivariate logistic regression was used to identify Desulfovibrionaceae, Parasutterella and TNA_a, Enterococcus, Parasutterella and MMIF. Parasutterella has a certain correlation with CA125 (**Supplementary Table 3**).

## Discussion

In this study, we compared gut microbiome structure, microbial abundance, species diversity, serum biomarkers, and clinical indicators between infertile women with endometriosis and those with infertility due to male factors. No statistically significant differences were observed between the two groups in terms of age, duration of infertility, anti-Müllerian hormone (AMH), or interleukin-6 (IL-6) levels (P > 0.05). However, women with endometriosis exhibited a lower body mass index (BMI) and higher serum CA125 levels compared to the control group (P < 0.05), consistent with previous reports (Rokhgireh et al., 2020; Chen et al., 2021; Rowlands et al., 2022). Notably, serum levels of macrophage migration inhibitory factor (MIF) and tumor necrosis factor-alpha (TNF-α) were significantly elevated in the endometriosis group, indicating their potential involvement in disease pathogenesis. MIF contributes to immune cell regulation and inflammatory responses, while TNF-α is a pro-inflammatory cytokine commonly associated with chronic inflammation in endometriosis. Elevated levels of these biomarkers may exacerbate tissue injury and support the growth of ectopic endometrial lesions (Zhang et al., 2018; Tulandi and Vercellini, 2024). Thus, MIF and TNF-α represent promising targets for therapeutic intervention in endometriosis.

No significant differences in gut microbiota α-diversity or β-diversity were detected between the endometriosis and control groups. This may be attributed to the considerable heterogeneity among endometriosis patients and the fact that most existing studies focus on taxonomic abundance rather than diversity. Current evidence does not support a general reduction in gut microbial diversity in endometriosis. Moreover, the clinical relevance of microbial diversity remains debated, as higher diversity does not invariably confer better health outcomes. In fact, microbial diversity often declines following successful treatment of various diseases (Qin et al., 2022; Talwar et al., 2022; Li et al., 2025).

Emerging evidence suggests that endometriosis may be driven by chronic inflammation and systemic immune dysregulation (Greygoose et al., 2025). In our cohort, we observed a distinct gut microbial profile in endometriosis patients, characterized by enrichment of Lachnospira, Bacilli, Lactobacillales, Parasutterella, Enterococcus, and Veillonella. This compositional shift may be indicative of, or contribute to, an altered inflammatory and immune milieu, as supported by associations with inflammatory markers discussed below (Zhou et al., 2021).

Comparative analysis revealed significant between-group differences in the relative abundance of several bacterial taxa. Specifically, Lachnospira, Veillonella, and Streptococcus were more abundant in the endometriosis group, whereas Parasutterella, Alistipes, and Desulfovibrionaceae were more prevalent in controls. The direction of change for certain taxa diverges from reports in other cohorts. For example, while we observed an increase in Lachnospirain endometriosis patients, a recent study on gynecological surgery patients reported a decreasein Lachnospiraafter abdominal hysterectomy (Iavarone et al., 2023). This discrepancy may reflect differences in study populations (infertile endometriosis vs. surgical patients) or disease status, as surgery and associated physiological stress can markedly alter gut microbial composition—illustrated by the same study noting a postoperative increase in Proteobacteria. Conversely, the increased abundance of Alistipesand Rikenellaceae in our controls aligns with their reported association with gut ecosystem stabilityy (Pan et al., 2023). Although our study did not directly measure microbial metabolic output, the enrichment of Lachnospiraand Veillonellain patients remains noteworthy given their established roles in short-chain fatty acid and lactate metabolism, which could influence host inflammatory toney (Feng et al., 2018; Cong et al., 2022; Abdelhalim, 2024). Together, these comparisons highlight that microbial signatures in endometriosis may vary with clinical context, and underscore the importance of interpreting taxonomic changes in light of patient phenotype, interventions, and methodological approach.Correlation analysis further revealed several inter-species associations: Desulfovibrionaceaewas negatively correlated with Bilophila (P < 0.05) and positively correlated with Peptoniphilus (P < 0.05). Enterococcuswas positively correlated with Streptococcus (P < 0.05), and Ruminococcaceaeshowed a positive correlation with Bilophila (P < 0.05). These findings suggest that endometriosis-associated alterations in gut microbiota may arise from complex ecological interactions within the microbial community, rather than isolated changes in individual species.

More directly, our data link specific microbiota to systemic inflammatory markers.We identified a positive correlation between Streptococcusand Veillonellaand serum MIF levels, which were significantly elevated in the endometriosis group. MIF is a pluripotent immunoregulatory cytokine (Bucala, 2013; Sumaiya et al., 2022). Certain bacteria, including some Streptococcusstrains, can stimulate MIF secretion (Ge and Sun, 2014). The positive association observed here suggests a potential role for these bacteria in modulating host immune responses, though the precise mechanism requires further investigation.We also observed positive correlations of Bilophilaand Enterococcuswith TNF-α and IL-6, and of Veillonellawith TNF-α. These correlations are consistent with the pro-inflammatory potential attributed to these genera in other pathological contexts (Walker and Schmitt-Kopplin, 2021; Abulughod et al., 2024). In contrast, Desulfovibrionaceaewas negatively correlated with TNF-α and IL-6, which aligns with literature suggesting anti-inflammatory properties for metabolites produced by sulfate-reducing bacteria (Macfarlane and Macfarlane, 2012; Blachier et al., 2019; Munteanu et al., 2024). These correlative data collectively support a link between the endometriosis-associated gut microbiota and a pro-inflammatory systemic state, but do not establish causality or detail the underlying pathways.

A negative correlation was also observed between Parasutterellaand CA125, a biomarker elevated in endometriosis (Charkhchi et al., 2020). While this inverse association is novel, its biological significance remains unclear and may reflect an indirect relationship mediated by systemic inflammation associated with gut dysbiosis (Mostafavi Abdolmaleky and Zhou, 2024).

Based on the observed correlations, we hypothesize that the altered gut microbiota in endometriosis patients may contribute to, or be a consequence of, the chronic inflammatory and immune dysregulation characteristic of the disease. Modulating the gut microbiota could therefore represent a potential therapeutic avenue, a hypothesis that requires interventional studies for validation.

Another important consideration is the heterogeneity of endometriosis itself. Women in our cohort likely differed in terms of disease stage, lesion location (ovarian, peritoneal, deep infiltrating), and inflammatory phenotype. These factors may influence both local pelvic inflammation and systemic immune responses, and they could also be associated with distinct gut microbiota signatures. In the current pilot study, we did not have sufficient power to stratify analyses by detailed clinical subtypes. Future investigations with larger sample sizes and standardized phenotyping are needed to determine whether specific microbiome–immune patterns are associated with particular endometriosis phenotypes or with treatment response.

This study has several strengths. It focuses on a clearly defined cohort of infertile women with and without endometriosis, collected within a standardized menstrual cycle window. Recent use of antibiotics, probiotics, and immunosuppressive drugs was excluded to reduce overt perturbations of the gut microbiota. An ASV-based 16S rRNA sequencing pipeline with explicit quality control was applied, and raw data were deposited in a public repository to enhance reproducibility. Importantly, we integrated gut microbiota profiling with the measurement of multiple systemic immune-inflammatory markers, enabling exploration of microbiome–immune relationships within the same individuals.

This study has several limitations. First, it was an exploratory pilot investigation with a relatively small sample size from a single center, which limits statistical power and generalizability. Second, the control group consisted of women with male-factor infertility rather than strictly healthy women, and subclinical pelvic inflammation or microbiota alterations cannot be entirely excluded. Third, we relied on 16S rRNA gene sequencing of the V1–V2 region, which offers limited taxonomic resolution and no direct functional information; functional metagenomics and metabolomics were not performed. Fourth, although we attempted to minimize variability by standardizing the sampling window and excluding recent antibiotic or probiotic use, dietary patterns, physical activity, and detailed hormonal treatment history were not systematically recorded. Finally, the cross-sectional design precludes causal inference regarding the directionality of associations between gut microbiota, systemic inflammation, and infertility outcomes. Further mechanistic research is also needed to elucidate the pathways linking gut microbiota to inflammatory responses in infertile women with endometriosis.

## Conclusion

In conclusion, this exploratory study provides preliminary evidence of disruptions in the gut microbiome and metabolome in women with endometriosis-related infertility, linked to altered levels of MMIF, TNF-α, and IL-6. As an initial investigation, it suggests that gut dysbiosis may influence these key inflammatory factors through host-microbiome interactions and microbial metabolites, potentially contributing to the inflammatory pathology of endometriosis and associated infertility. While the exact mechanisms remain to be confirmed, these observations offer novel exploratory insights into the biological interplay between the gut ecosystem and reproductive-immune dysregulation in this context. Further research is warranted to validate and expand upon these preliminary findings.

## Data Availability

The data have been uploaded to the National Center for Biotechnology Information Sequence Read Archive and are accessible under the BioProject accession number PRJNA1359364.
