# Supplementary material for: Gut microbiota composition and systemic immune-inflammatory marker correlations in infertile women with endometriosis: a pilot case–control study
Source: Front Cell Infect Microbiol. 2026 Jan 23;16:1720894. doi: 10.3389/fcimb.2026.1720894 (PMC12876246; doi:10.3389/fcimb.2026.1720894)
Supplement: Supplementary file 1 [file Table1.docx]

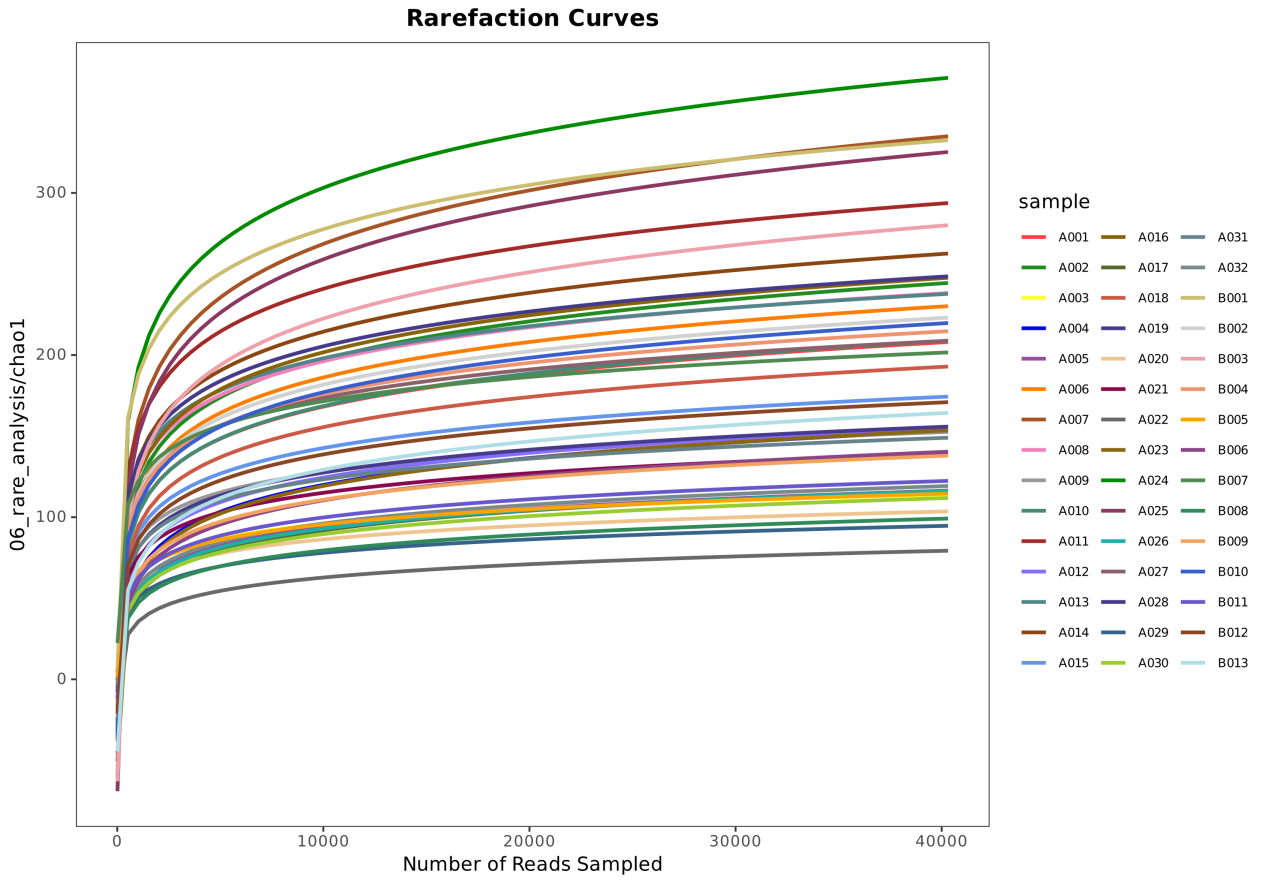


**Supplementary Figure 1.** Rarefaction curves of sampling sequencing depth.

**Supplementary Table S1.** LEfSe Specific Information Table.

| tax | Sign_Group | LDA | FDR | EMs-group_mean | Normal-group_mean | Total mean |
| --- | --- | --- | --- | --- | --- | --- |
| f__Rikenellaceae | Normal-group | 3.400289159 | 0.027992726 | 0.630245599 | 1.136391771 | 0.776465604 |
| g__Alistipes | Normal-group | 3.400522348 | 0.027992726 | 0.629936124 | 1.136391771 | 0.776245533 |
| s__un_g_Alistipes | Normal-group | 3.400522348 | 0.027992726 | 0.629936124 | 1.136391771 | 0.776245533 |
| c__Bacilli | EMs-group | 3.703283628 | 0.016762181 | 1.229853185 | 0.208157568 | 0.934696673 |
| o__Lactobacillales | EMs-group | 3.700695324 | 0.014610418 | 1.221419995 | 0.206443553 | 0.928204578 |
| f__Enterococcaceae | EMs-group | 3.639413528 | 0.032759388 | 0.918830928 | 0.001142676 | 0.653720988 |
| g__Enterococcus | EMs-group | 3.606896808 | 0.031088629 | 0.91867619 | 0.00095223 | 0.653555935 |
| s__un_g_Enterococcus | EMs-group | 3.622994054 | 0.031088629 | 0.91867619 | 0.00095223 | 0.653555935 |
| f__Streptococcaceae | EMs-group | 2.762208681 | 0.004137658 | 0.219649798 | 0.102459992 | 0.185794965 |
| g__Streptococcus | EMs-group | 2.764737434 | 0.003821708 | 0.219417692 | 0.101507762 | 0.185354823 |
| s__un_g_Streptococcus | EMs-group | 2.764737434 | 0.003821708 | 0.219417692 | 0.101507762 | 0.185354823 |
| g__Lachnospira | EMs-group | 4.010161924 | 0.003507212 | 2.408952489 | 0.503729887 | 1.858554849 |
| s__un_g_Lachnospira | EMs-group | 4.010161924 | 0.003507212 | 2.408952489 | 0.503729887 | 1.858554849 |
| f__Veillonellaceae | EMs-group | 3.659465032 | 0.008538677 | 1.340103612 | 0.507348362 | 1.099529873 |
| g__Veillonella | EMs-group | 3.381176592 | 0.001208363 | 0.538408928 | 0.052563118 | 0.398053472 |
| s__un_g_Veillonella | EMs-group | 3.381176592 | 0.001208363 | 0.538408928 | 0.052563118 | 0.398053472 |
| g__Bilophila | EMs-group | 2.74508453 | 0.046639221 | 0.126188384 | 0.012188549 | 0.093255098 |
| g__Bilophila\|s__un_g_Bilophila | EMs-group | 2.756376268 | 0.046639221 | 0.126188384 | 0.012188549 | 0.093255098 |
| g__un_f_Desulfovibrionaceae | Normal-group | 3.255204485 | 0.000267416 | 0.055396004 | 0.374988335 | 0.147722677 |
| s__un_f_Desulfovibrionaceae | Normal-group | 3.254516346 | 0.000267416 | 0.055396004 | 0.374988335 | 0.147722677 |
| g__Parasutterella | EMs-group | 3.498318787 | 0.024438455 | 1.419329182 | 1.390065951 | 1.41087536 |
| s__un_g_Parasutterella | EMs-group | 3.498318787 | 0.024438455 | 1.419329182 | 1.390065951 | 1.41087536 |

**Supplementary Table 2.** Original p-values and FDR correction for species tested by Wilcoxon rank-sum test.

| tax | p | after fdr |
| --- | --- | --- |
| g__Peptoniphilus | 0.00028 | 0.0028 |
| g__Streptococcus | 0.00130 | 0.0065 |
| g_Alistipes | 0.00370 | 0.0123 |
| g__un_f_Desulfovibrionaceae | 0.00400 | 0.0100 |
| g__Lachnospira | 0.02500 | 0.0500 |
| g__Veillonella | 0.02700 | 0.0450 |
| g__Parasutterella | 0.02900 | 0.0414 |
| g__Bilophila | 0.03200 | 0.0400 |
| g_Ruminococcaceae_UCG-009 | 0.04500 | 0.0500 |
| g__Enterococcus | 0.04800 | 0.0480 |

**Supplementary Table 3.** Multivariate logistic regression analysis of MMIF, TNF-α, IL-6, CA125 and gut microbiota.

|  | | | | | | | | | | | | | | | | |
| --- | --- | --- | --- | --- | --- | --- | --- | --- | --- | --- | --- | --- | --- | --- | --- | --- |
| Variables | TNF_a | | | | IL_6 | | | | MMIF | | | | CA125 | | | |
|  | Model1 | | Model2 | | Model1 | | Model2 | | Model1 | | Model2 | | Model1 | | Model2 | |
|  | OR (95%CI) | P | OR (95%CI) | P | OR (95%CI) | P | OR (95%CI) | P | OR (95%CI) | P | OR (95%CI) | P | OR (95%CI) | P | OR (95%CI) | P |
| g__Bilophila |  |  |  |  |  |  |  |  |  |  |  |  |  |  |  |  |
| Negative | 1.00 (Reference) |  | 1.00 (Reference) |  | 1.00 (Reference) |  | 1.00 (Reference) |  | 1.00 (Reference) |  | 1.00 (Reference) |  | 1.00 (Reference) |  | 1.00 (Reference) |  |
| Positive | 2.36 (0.67 ~ 8.27) | 0.179 | 2.23 (0.59 ~ 8.44) | 0.237 | 1.58 (0.46 ~ 5.41) | 0.464 | 1.60 (0.43 ~ 5.99) | 0.486 | 1.07 (0.32 ~ 3.63) | 0.912 | 0.79 (0.20 ~ 3.09) | 0.733 | 0.49 (0.14 ~ 1.70) | 0.26 | 0.38 (0.10 ~ 1.47) | 0.163 |
| g__un_f_Desulfovibrionaceae |  |  |  |  |  |  |  |  |  |  |  |  |  |  |  |  |
| Negative | 1.00 (Reference) |  | 1.00 (Reference) |  | 1.00 (Reference) |  | 1.00 (Reference) |  | 1.00 (Reference) |  | 1.00 (Reference) |  | 1.00 (Reference) |  | 1.00 (Reference) |  |
| Positive | 3.28 (0.97 ~ 11.13) | 0.056 | 4.30 (1.05 ~ 17.66) | 0.043 | 4.90 (1.39 ~ 17.31) | 0.014 | 5.12 (1.27 ~ 20.61) | 0.022 | 0.76 (0.24 ~ 2.47) | 0.652 | 0.61 (0.16 ~ 2.31) | 0.467 | 1.56 (0.48 ~ 5.06) | 0.459 | 1.45 (0.42 ~ 5.04) | 0.56 |
| g__Streptococcus |  |  |  |  |  |  |  |  |  |  |  |  |  |  |  |  |
| High | 1.00 (Reference) |  | 1.00 (Reference) |  | 1.00 (Reference) |  | 1.00 (Reference) |  | 1.00 (Reference) |  | 1.00 (Reference) |  | 1.00 (Reference) |  | 1.00 (Reference) |  |
| Low | 1.88 (0.57 ~ 6.14) | 0.297 | 2.39 (0.63 ~ 9.08) | 0.2 | 1.31 (0.41 ~ 4.23) | 0.652 | 1.59 (0.44 ~ 5.75) | 0.482 | 0.92 (0.28 ~ 2.95) | 0.884 | 1.00 (0.27 ~ 3.73) | 0.996 | 0.45 (0.13 ~ 1.47) | 0.184 | 0.44 (0.12 ~ 1.55) | 0.202 |
| g__Veillonella |  |  |  |  |  |  |  |  |  |  |  |  |  |  |  |  |
| High | 1.00 (Reference) |  | 1.00 (Reference) |  | 1.00 (Reference) |  | 1.00 (Reference) |  | 1.00 (Reference) |  | 1.00 (Reference) |  | 1.00 (Reference) |  | 1.00 (Reference) |  |
| Low | 0.92 (0.28 ~ 2.95) | 0.884 | 0.81 (0.22 ~ 3.00) | 0.75 | 1.31 (0.41 ~ 4.23) | 0.652 | 1.85 (0.48 ~ 7.10) | 0.37 | 0.30 (0.09 ~ 1.03) | 0.056 | 0.37 (0.09 ~ 1.42) | 0.147 | 0.30 (0.09 ~ 1.03) | 0.056 | 0.29 (0.08 ~ 1.09) | 0.067 |
| g__Enterococcus |  |  |  |  |  |  |  |  |  |  |  |  |  |  |  |  |
| Negative | 1.00 (Reference) |  | 1.00 (Reference) |  | 1.00 (Reference) |  | 1.00 (Reference) |  | 1.00 (Reference) |  | 1.00 (Reference) |  | 1.00 (Reference) |  | 1.00 (Reference) |  |
| Positive | 1.90 (0.56 ~ 6.46) | 0.301 | 2.34 (0.57 ~ 9.60) | 0.236 | 1.30 (0.39 ~ 4.34) | 0.672 | 1.19 (0.30 ~ 4.68) | 0.807 | 6.86 (1.74 ~ 27.08) | 0.006 | 7.91 (1.56 ~ 40.08) | 0.013 | 1.30 (0.39 ~ 4.34) | 0.672 | 1.22 (0.33 ~ 4.56) | 0.769 |
| g__Parasutterella |  |  |  |  |  |  |  |  |  |  |  |  |  |  |  |  |
| Negative | 1.00 (Reference) |  | 1.00 (Reference) |  |  |  | 1.00 (Reference) |  | 1.00 (Reference) |  | 1.00 (Reference) |  | 1.00 (Reference) |  | 1.00 (Reference) |  |
| Positive | 0.22 (0.05 ~ 0.95) | 0.043 | 0.22 (0.04 ~ 1.14) | 0.071 | 0.60 (0.16 ~ 2.27) | 0.447 | 0.52 (0.11 ~ 2.58) | 0.426 | 0.11 (0.02 ~ 0.61) | 0.011 | 0.13 (0.02 ~ 0.82) | 0.03 | 0.11 (0.02 ~ 0.61) | 0.011 | 0.10 (0.02 ~ 0.62) | 0.013 |
| OR: Odds Ratio, CI: Confidence Interval Model1: Crude Model2: Adjust: BMI, years, AMH, Age | | | | | | | | | | | | | | | | |
